# Interactions between *Leishmania braziliensis* and Macrophages Are Dependent on the Cytoskeleton and Myosin Va

**DOI:** 10.1155/2012/275436

**Published:** 2012-06-27

**Authors:** Elisama Azevedo, Leandro Teixeira Oliveira, Ana Karina Castro Lima, Rodrigo Terra, Patrícia Maria Lourenço Dutra, Verônica P. Salerno

**Affiliations:** ^1^Laboratório de Imunologia e Bioquímica de Protozoários, Departamento de Microbiologia, Imunologia e Parasitologia, FCM, UERJ, Avenida Professor Manuel de Abreu 444 5° andar. Vila Isabel, 20550-170 Rio de Janeiro, RJ, Brazil; ^2^Programa de Pós-Graduação em Microbiologia Médica, Faculdade de Ciências Médicas, UERJ, 20550-170 Rio de Janerio, RJ, Brazil; ^3^Departamento Biociências, Escola de Educação Física e Desportos, Universidade Federal do Rio de Janeiro, 21941-599 Rio de Janerio, RJ, Brazil; ^4^Programa de Pós-Graduação em Biodinâmica do Movimento, EEFD, UFRJ, 21941-599 Rio de Janerio, RJ, Brazil

## Abstract

Leishmaniasis is a neglected tropical disease with no effective vaccines. Actin, microtubules and the actin-based molecular motor myosin Va were investigated for their involvement in *Leishmania braziliensis* macrophage interactions. Results showed a decrease in the association index when macrophages were without F-actin or microtubules regardless of the activation state of the macrophage. In the absence of F-actin, the production of NO in non-activated cells increased, while in activated cells, the production of NO was reduced independent of parasites. The opposite effect of an increased NO production was observed in the absence of microtubules. In activated cells, the loss of cytoskeletal components inhibited the release of IL-10 during parasite interactions. The production of IL-10 also decreased in the absence of actin or microtubules in non-activated macrophages. Only the disruption of actin altered the production of TNF-**α** in activated macrophages. The expression of myosin Va tail resulted in an acute decrease in the association index between transfected macrophages and *L. braziliensis* promastigotes. These data reveal the importance of F-actin, microtubules, and myosin-Va suggesting that modulation of the cytoskeleton may be a mechanism used by *L. braziliensis* to overcome the natural responses of macrophages to establish infections.

## 1. Introduction

Leishmaniasis is caused by several different species of protozoan parasites from the genus *Leishmania*. *Leishmania* parasites maintain a life cycle consisting of a phase in a dipteran insect (sandflies) and a phase in a mammalian host. Transmission occurs when an infected sandfly bites a human. This can lead to infection of macrophages, in which the parasite thrives inside the hostile environment of the phagolysosomes [1]. Leishmaniasis is one of the most important of the neglected tropical diseases, with 350 million people in 88 countries worldwide living at risk of developing one of the many forms of the disease [2].

The numerous forms of leishmaniasis are dependent on factors that are not well understood, including the species of the parasite and the health of the host upon initial infection. The parasitosis can vary from self-healing dermal lesions to generalised organ infection, which can lead to death. *Leishmania braziliensis* is the causative agent of mucocutaneous disease in the Americas. Despite its great importance, it has been less studied than other strains because of difficulties in *in vitro* cultivation [3, 4].

The *Leishmania* parasites display multiple forms that are distinct in morphology, biochemistry, intracellular organisation, and behaviour. In the sandfly, the replicating form of *Leishmania* spp., the promastigote, is flagellated and motile. A subset of promastigotes progress through differentiation to become the nondividing, infectious metacyclic promastigotes. Following a bite by the sandfly, these metacyclic promastigotes are transmitted to the mammalian host. The process of infection begins when the parasites undergo conventional phagocytosis by macrophages that are recruited to the site of the bite. After phagocytosis, the parasites are located within classic phagolysosomes and undergo differentiation into the amastigote form, which is resistant to the acidic pH and lysosomal enzymes present in these cellular structures [5]. Amastigotes do not have an exterior flagellum and live as intracellular parasites in a variety of mammalian cells, most notably within professional phagocytes such as macrophages [6].

 Phagocytosis occurs by the extension of the plasma membrane around an extracellular particle, followed by internalisation of the particle into a membrane-bounded intracellular vesicle, the phagosome. In macrophages, different cell surface receptors stimulate various types of phagocytic responses [7]. Macrophage Fc receptors mediate the phagocytosis of IgG-coated particles. Ligation of Fc receptors initiates an intracellular signalling cascade that ultimately impinges on the actin cytoskeleton [8]. With regards to *Leishmania*, the phagocytic response is coupled to other cellular events that prevent the activation of deadly antimicrobial agents such as nitric oxide (NO) and many of the cytokine-inducible macrophages, which are necessary for the development of an effective immune response. This enables the parasite to evade the innate immune response and to divide within the phagolysosome of the infected macrophage, from whence it can spread and propagate the disease within the host [9].

It is well known that lipopolysaccharide (LPS) and IFN-*γ* promote classical macrophage activation (i.e., the activation of M1 macrophages). The phenotype of M1 macrophages includes high production of IL-12 and IL-23 and low production of IL-10, an anti-inflammatory cytokine. These cells are able to produce effectors molecules, such as reactive species of oxygen and nitric oxide (NO), and inflammatory cytokines, such as IL-1*β*, TNF-*α* and IL-6. M1 macrophages function as cellular immune response inductors, promoting T helper 1 lymphocyte (Th1) differentiation, and mediate resistance to intracellular pathogens and tumour cells [10].

The resistance or susceptibility to all forms of leishmaniasis has been associated to a balance between cellular and humoral immunity responses [11]. Several studies on *L. major* using models of infection in BALB/c and C57/BL6 mice have shown a good prognosis associated with immune responses that are predominantly Th1 as determined by the production levels of IL-12 and TNF-*α* cytokines. Susceptibility to more serious manifestations of leishmaniasis was associated with the activation of T helper 2 lymphocytes (Th2) based on the production of the anti-inflammatory cytokines IL-4 and IL-10 [12–16]. In cases of leishmaniasis caused by *L. braziliensis*, a major difference was observed. An exacerbated Th1 response (hiperergy) can be observed that promotes an increase in tissue damage near regions displaying high levels of parasitic antigens. This observation is characteristic of a classical mucosal leishmaniasis that is associated with high levels of IFN-*γ* and TNF-*α* and low levels of IL-10. In addition, cells collected from individuals presenting this type of leishmaniasis display a poor response to cytokines that inhibit IFN-*γ* secretion [17].

There are no effective vaccines available for leishmaniasis, and treatments rely on parenteral drugs that present high toxicity, low efficacy, and, in some cases, widespread resistance [18, 19]. The most common drugs are nephro-, hepato-, and cardiotoxic [20]. Investigative studies of the relationship between *Leishmania* and its host cells are imperative for identifying potential targets that can interfere with the process of invasion. Numerous studies have shown that interference with the parasite-host interaction can be effective. For example, a decrease in the association index between *Leishmania* and macrophages was observed when 4,5,6,7-tetrabromobenzotriazole (TBB), a specific casein kinase 2 (CK2) inhibitor, was used [21]. TBB was also able to reverse the positive platelet-activating factor (PAF) effect on this type of cellular interaction [22]. A similar profile of inhibition was reported using 1,10-phenanthroline (phen) and 1,10-phenanthroline-5,6-dione (phendio), which are ion chelators that inhibit metal-dependent peptidases [23].

TBB also induced changes in cell shape and the cytoskeleton, which are important to the process of phagocytosis. Particle ingestion by phagocytosis results from sequential rearrangements of the actin cytoskeleton and the overlying membrane [24]. Inhibitors and/or enzymes capable of interfering with the dynamics of cytoskeletal components, such as latrunculin A, which acts specifically to disrupt the actin cytoskeleton [25], and nocodazole, which depolymerises microtubules [26], are good candidates for the study of parasite-host cell interactions. In addition, the molecular motor myosin Va has been observed to be associated with phagosomes [27].

Latrunculin A (2-thiazolidinone macrolide) is a toxin purified from the red sea sponge *Latrunculia magnifica*. This substance sequesters G-actin and prevents F-actin assembly. It binds monomeric actin with 1 : 1 stoichiometry and can be used to block the polymerisation of purified actin (*Kd = 0.2 *μ*M*). The effects of this toxin are observed in cell cultures when used at a range of 0.1 and 10 *μ*M [28, 29]. These effects appear to occur rapidly, with the toxin promoting the depolymerisation of tumour cell cytoskeletons within 10 minutes [29].

Nocodazol is an antimitotic agent that disrupts microtubules by binding to *β*-tubulin [30], thereby promoting the inhibition of microtubule dynamics. These effects can be observed in cells after 5 minutes of treatment [31]. Nocodazol promotes disruption of the mitotic spindle [31, 32] and fragmentation of the Golgi complex [33].

In the present study, we investigated the involvement of actin and the actin-based molecular motor myosin Va in the interaction between *Leishmania braziliensis* and macrophages.

## 2. Material and Methods

### 2.1. Chemicals

 5-(and-6)-Carboxyfluorescein diacetate, succinimidyl ester (green CFSE), chloromethyl tetramethylrhodamine (orange CMTMR), and latrunculin A were purchased from Invitrogen (Eugene, Oregon, USA). Foetal calf serum (FCS) was purchased from Cultilab Co (Campinas, São Paulo, Brazil). Nocodazol and all other chemicals used in this work were purchased from Sigma (St. Louis, MO, USA).

### 2.2. Parasites 

The strain *L. (V.) braziliensis *(MHOM/BR/2002/EMM-IOC-L2535) was maintained in the promastigote form by culturing at 26°C in Schneider's medium supplemented with 2 mM glutamine, 100 units/mL penicillin, 100 mg/mL streptomycin, and 10% FCS. For the interaction assays, the promastigotes were first labelled by incubation with 5 *μ*M green CFSE for 10 min at 37°C or with 5 *μ*M orange CMTMR for 20 min, followed by two washes with PBS by centrifugation, and the final pellet was suspended in Dulbecco's Modified Eagle Medium (DMEM) supplemented with 10% FCS.

### 2.3. Cell Culture 

The RAW 264.7 macrophage cell line (kindly supplied by Dr. Marcia Paes—Laboratório de Bioquímica, Universidade do Estado do Rio de Janeiro, UERJ, Rio de Janeiro, Brazil) was maintained at 37°C in DMEM medium supplemented with 10% foetal calf serum, penicillin (100 units/mL), and streptomycin (100 mg/mL) in a humidified atmosphere of 4% CO_2_.

### 2.4. *Leishmania braziliensis*-Macrophages Interactions

 Macrophages were seeded onto 24-well plates containing glass coverslips for 2 h at 37°C in a 4% CO_2_ atmosphere, washed once with DMEM and incubated for 17 h in the presence or absence of lipopolysaccharide (LPS) (100 ng/mL) and INF-*γ* (1 *μ*g/mL) at 37°C in a 4% CO_2_ atmosphere, as previously described [34, 35]. At the moment of the experiment, the culture medium was replaced with DMEM containing either latrunculin A (5 *μ*M), nocodazole (5 *μ*M), or no drug at 37°C in a humidified atmosphere of 4% CO_2_ for 10 minutes. Then, promastigotes that had been previously marked with CSFE were added and allowed to interact with the macrophages at a 5 : 1 ratio for 30 min at 37°C in a 4% CO_2_ atmosphere. After incubation, the coverslips were washed 3 times with PBS, fixed and stained with Giemsa, and the percentage of infected macrophages was determined by counting 600 cells in triplicate coverslips, as described. The association index was determined by multiplying the percentage of infected macrophages (cells with at least one associated or intracellular parasite) by the mean number of parasites per cell (internalised or simply attached to the cell) [36]. The time of latrunculin and nocodazole incubation used in this work were chosen in accordance to the cellular viability test. This time did not affect the macrophages viability.

For myosin Va detection and actin staining, the parasites were treated with 5 *μ*M green CFSE or with 5 *μ*M orange CMTMR (only in the case of myosin Va) and allowed to interact with the macrophages under the conditions described above. After interaction, the cells were fixed with 4% paraformaldehyde, permeabilised with 0.1% Triton X-100, and stained with 1 : 250 Phalloidin Alexa 546 for 30 min at room temperature (for actin staining) or blocked using 1% bovine serum albumin (BSA) and 20% sheep serum (for myosin Va detection). These cells were incubated for 1 h with rabbit antimyosin Va primary antibodies (1 : 1000—Santa Cruz Biotech) and washed with PBS. The secondary antibody, anti-rabbit IgG conjugated to Alexa-546 (1 : 1000—Molecular Probes), was added. The cells used for myosin Va detection were placed on glass slides using *ProLong Antifade* mounting media (Molecular Probes). Coverslips containing the cells used for actin staining were treated with a saturated solution of n-propyl-gallate in PBS, followed by mounting on glass slides for microscopy. The images were collected by a Confocal Zeiss microscope (LSM 510 Meta, Zeiss, Germany) (software Zeiss Vision Release 4.6).

### 2.5. Nitrite Determination

 The nitrite that had accumulated in culture medium was measured as an indicator of NO production based on the Griess reaction. The macrophages were allowed to adhere to 24-well culture plates for 2 h at 37°C in a 4% CO_2_ atmosphere. Adherent cells were washed with DMEM and incubated for 17 h in fresh medium in the presence or absence of LPS (100 ng/mL) and IFN-*γ* (1 *μ*g/mL), as previously described [34, 35]. Next, the culture medium was removed, and the macrophages were incubated with latrunculin (5 *μ*M), nocodazole (5 *μ*M), or DMEM for 40 min at 37°C in a 4% CO_2_ atmosphere. The time of latrunculin and nocodazole incubation used in this work were chosen in accordance to the cellular viability test. This time did not affect the macrophages viability. For the *Leishmania*-macrophage interaction experiments, 10 min after the addition of latrunculin A and nocodazole, the promastigotes were washed with PBS and allowed to interact with the macrophages at a 5 : 1 ratio for 30 min at 37°C in a 4% CO_2_ atmosphere. In all cases, a sample of the supernatant was removed to measure the nitrite content by the Griess method [37]. Briefly, 50 *μ*L of cell culture medium was mixed with 50 *μ*L of Griess reagent and incubated at room temperature for 10 min, and then the absorbance was measured at 540 nm in a microplate reader (TP reader Thermo Plate). Fresh culture medium was used as the blank in all experiments. The amount of nitrite in the samples was calculated with a standard curve obtained from a serial dilution of sodium nitrite.

### 2.6. Cytokine Analysis

 Macrophages were seeded onto 24-well culture plates and allowed to adhere for 2 h at 37°C in a 4% CO_2_ atmosphere, washed in DMEM and incubated for 17 h in this medium in the presence of LPS (100 ng/mL) and IFN-*γ* (1 *μ*g/mL) at 37°C in a 4% CO_2_ atmosphere, as previously described [34, 35]. Next, latrunculin (5 *μ*M) or nocodazole (5 *μ*M) was added directly to the incubation medium, and the cells were incubated for 1 h at 37°C in a 4% CO_2_ atmosphere before the addition of parasites. The time of latrunculin and nocodazole incubation used in this work were chosen in accordance to the cellular viability test. This time did not affect the macrophages viability. The supernatant of macrophages was recovered and used to resuspend the promastigotes, and this suspension was added to the macrophages at a ratio of 5 : 1 (parasites : macrophages). These cells were allowed to interact for 30 min at 37°C in a 4% CO_2_ atmosphere. The supernatants were recovered for IL-10 or TNF-*α* measurements. Uninfected macrophages that were treated in the same manner were used as controls. All supernatants used to measure the cytokines were the result of 18 h and 30 min incubation. The level of cytokines was determined in the supernatants by ELISA (R&D Systems, USA) using recombinant murine cytokines and antibodies according to the manufacturer's instructions. The absorbance was measured at 450 nm in a microplate reader (TP reader Thermo Plate). The cytokine concentrations were evaluated using IL-10 and TNF-*α* standard curves.

### 2.7. Cellular Viability Test

Macrophages treated with latrunculin A and nocodazole for 1 hour and 30 minutes (the maximal incubation time used in this work) had their cellular viability assessed byan MTT [3-(4,5-dimethylthiazol-2-yl)-2,5-diphenyl tetrazolium bromide] assay as previously described [38].

### 2.8. Transfection of Macrophages with eGFP-DB (Myosin Va Brain-Isoform Tail) for *Leishmania braziliensis*-Macrophage Interaction Assays 

The macrophages were seeded onto poly-L-lysine-coated coverslips in 24-well plates in DMEM without antibiotics. Lipofectamine 2000 was diluted into 25 *μ*L of Opti-MEM for 5 minutes at room temperature and then mixed with plasmid DNA that had been diluted in Opti-MEM for 20 min. After this period of incubation, the DMEM was removed from the macrophage cultures, and the transfection mixture was layered on top of the cells. The cells were incubated for 1 h at 37°C in a humidified atmosphere of 5% CO_2_, and then 1 mL DMEM supplemented with 10% foetal calf serum was added. The cells were incubated for an additional 18 h at 37°C in a humidified atmosphere of 5% CO_2_ to permit expression from the plasmids. Subsequently, an interaction assay was carried out as previously described. The cells were observed on a Zeiss confocal microscope (LSM 510 Meta, Zeiss, Germany) and imaged using the Zeiss Vision Release 4.6 software.

### 2.9. Statistical Analysis 

All results are presented as the mean and standard error of the mean (SEM). Normalised data were analysed by a one-way analysis of variance (ANOVA), and differences between groups were assessed with the Tukey posttest. We used the software GraphPad Prism 4.0, and values of *P* ≤ 0.05 were considered significant.

## 3. Results

### 3.1. Effect of Latrunculin and Nocodazole on the Interaction of *Leishmania braziliensis* with RAW 264.7 Macrophages Activated by LPS and IFN-*γ*

**Figure 1:**
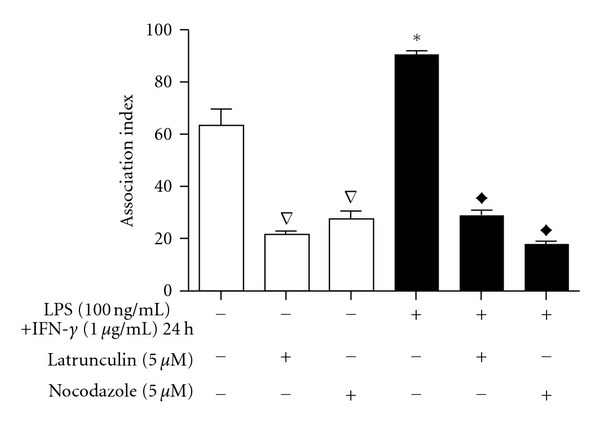
Effect of latrunculin and nocodazole on the interaction of *Leishmania braziliensis *with RAW 264.7 macrophages. Adherent macrophages, with or without 17 h previous activation by LPS and IFN-*γ*, were treated with latrunculin (5 *μ*M) or nocodazole (5 *μ*M) for 10 min and then incubated with promastigotes for 30 min. The association indices were determined by multiplying the percentage of infected macrophages by the mean number of parasites per cell. The bars represent the mean ± standard errors of mean (SEM) of at least three independent experiments performed in triplicate. *P ≤ 0.05 in relation to control inactivated macrophages, ^Δ^ *P* ≤ 0.05 in relation to control inactivated macrophages,^*◆*^ *P* ≤ 0.05 in relation to control activated macrophages.

Studies with other *Leishmania* strains have suggested that the cytoskeleton is involved in the interactions between the parasite and the host macrophages. However, the different strains also exhibit variations in the dynamics of cell invasion. To understand the role of the actin and microtubule cytoskeleton of macrophages during interactions with *L. braziliensis*, the cytoskeleton-disrupting drugs latrunculin A [25] and nocodazole [26] were used to depolymerise filamentous actin and microtubules, respectively. The cellular viability of macrophages was not altered by drug treatment (data not shown). The drugs were added 10 min before the addition of parasites to prevent artefacts arising from the kinetics of depolymerisation. In the absence of F-actin, the association index of parasites to macrophages decreased 66.45% when compared to the nontreated controls. The absence of microtubules decreased the association index by 57.04% (Figure 1).

Because of the high potential for parasites to interact with activated macrophages *in vivo*, similar experiments were performed after 24 h of stimulating macrophages with LPS (100 ng/mL) and IFN-*γ* (1.0 *μ*g/mL). Activated macrophages (M1) that were not treated with the cytoskeleton-disrupting agents showed a significant increase in the association index compared to the nonactivated macrophages. The loss of both F-actin and microtubules decreased the association index significantly. In the presence of latrunculin A, the association index between *L. braziliensis* and M1 macrophages was inhibited by 68.71%, while in the absence of microtubules as a result of nocodazole treatment, the inhibition was 80.17% (Figure 1).

### 3.2. Effect of Latrunculin and Nocodazole on NO Production by RAW 264.7 Macrophages

**Figure 2:**
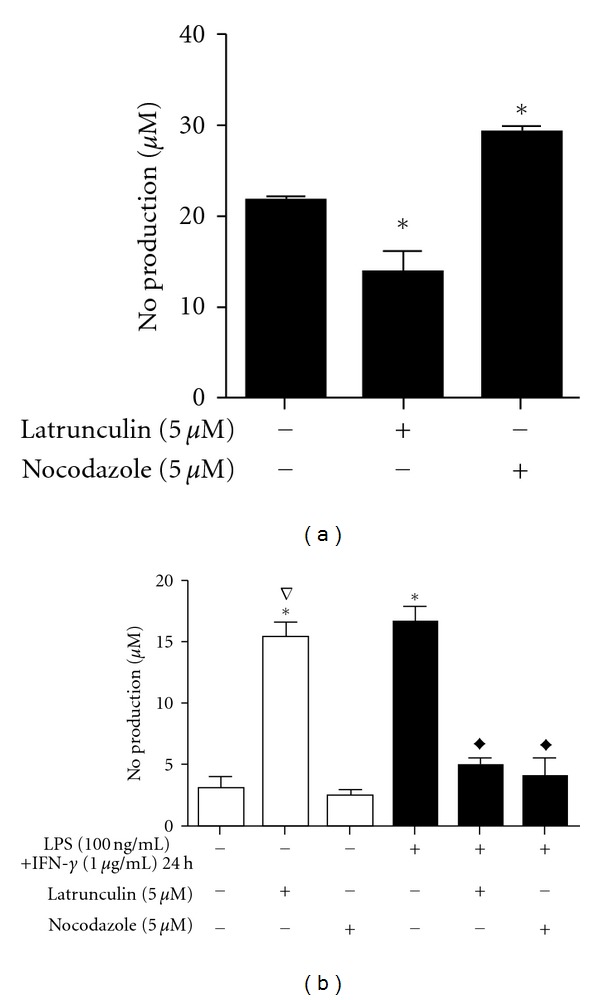
Effect of latrunculin and nocodazole on the production of NO by RAW 264.7 macrophages after interaction with *L. brasiliensis*. NO levels were inferred from the level of nitrite measured in the media after various treatments. Adherent macrophages were activated (black bars) for 17 h with LPS (100 ng/mL) + IFN-*γ* (1 *μ*g/mL) or remained untreated (open bars). For the measurement of NO after interactions with parasites in (a), macrophages were treated with latrunculin (5 *μ*M) or nocodazole (5 *μ*M) for 10 min and then exposed to promastigotes for 30 min. In the absence of parasites (b), activated and nonactivated adherent macrophages were treated with latrunculin (5 *μ*M) or nocodazole (5 *μ*M) for 10 min. The bars represent the mean ± standard errors of mean (SEM) of at least three independent experiments performed in triplicate. *P ≤ 0.05 compared to control activated macrophages in (a) and compared to control inactivated macrophages in (b); ^∇^ *P* ≤ 0.05 compared to control nonactivated macrophages; ^*◆*^ *P* ≤ 0.05 compared to control activated macrophages.

NO production is the main leishmanicidal process performed by macrophages, and the *Leishmania* genus has developed evasion mechanisms that interfere with NO production [39]. To determine the influence of the cytoskeleton on the production of NO by macrophages, nitrite was measured following disruption of F-actin and microtubules before and after interactions with the parasites (Figure 2). In control M1 macrophages, the presence of *L. braziliensis* promoted a small increase (approximately 20%) in the level of NO (Figure 2(a)) when compared to uninfected M1 macrophages (Figure 2(b)). The NO production in response to the parasites decreased by 36.86% after latrunculin A treatment. Treatment with nocodazole had the opposite effect, as the level of NO production increased by 36.17% (Figure 2(a)).

The effect of cytoskeleton disruption was dependent on the activation state of macrophages in addition to the presence of parasites. The NO production in nonactivated macrophages was significantly increased by latrunculin treatment (390%), reaching the same levels as that produced by the M1 macrophages. Conversely, latrunculin A treatment decreased NO production by 70% in activated cells. Nocodazole had no effect in non-activated cells, while in M1 macrophages, this substance inhibited NO production by 75% (Figure 2(b)).

### 3.3. Effect of Latrunculin and Nocodazole on the Production of IL-10 and TNF-*α* Cytokines by RAW 264.7 Macrophages

**Figure 3:**
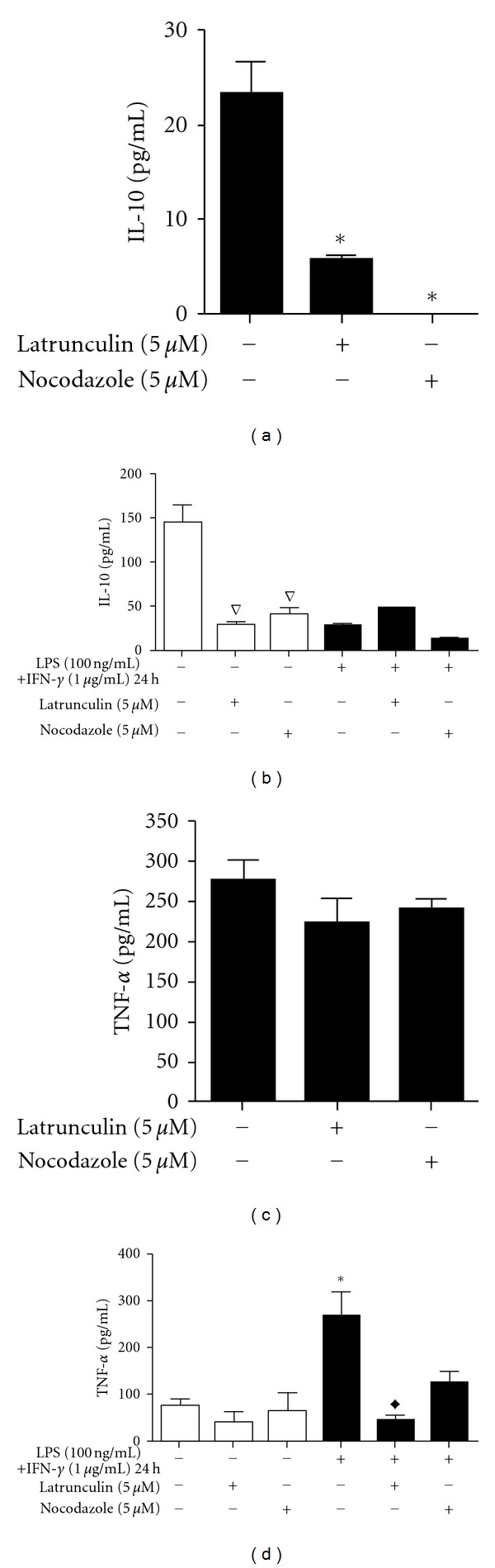
Effect of latrunculin and nocodazole on cytokine production in RAW 264.7 macrophages. Levels of IL-10 (a and b) and TNF-*α* (c and d) were measured in the media after various treatments. Adherent macrophages were treated with LPS (100 ng/mL) + IFN-*γ* (1 *μ*g/mL) for 17 h before treatment with latrunculin (5 *μ*M) or nocodazole (5 *μ*M) for 1 h and then exposed to promastigotes for 30 min (a and c). Adherent macrophages not exposed to parasites were incubated for 17 h in the absence or in the presence of LPS (100 ng/mL) plus IFN-*γ* (1 *μ*g/mL) and then treated with latrunculin (5 *μ*M) or nocodazole (5 *μ*M) for 1 h (b and d). The bars represent the mean ± standard errors of mean (SEM) of at least three independent experiments performed in triplicate. *P ≤ 0.05 compared to control infected macrophages (a) and compared to control non-activated and uninfected macrophages; ^∇^ *P* ≤ 0.05 compared to control non-activated and uninfected macrophages; ^*◆*^ *P* ≤ 0.05 compared to control activated uninfected macrophages.

Cytokine modulation is another macrophage response to activation or the internalisation of pathogens. In this work, the effect of the release of IL-10 and TNF-*α* on the cytoskeleton and interactions with parasites was evaluated. We observed an inhibition of IL-10 release by M1 macrophages, and interactions with *L. braziliensis* promoted a further decrease in IL-10 release (Figures 3(a) and 3(b)). Treatment with latrunculin during parasite interactions with M1 macrophages reduced the release of IL-10 by 79.17%. Nocodazole treatment had a greater effect and abrogated IL-10 release. Similar results were observed in non-activated macrophages in the absence of parasite interactions (Figure 3(b)). Latrunculin and nocodazole were able to decrease the release of IL-10 by non-activated macrophages by 84.5% and 83%, respectively. No significant changes were detected in uninfected M1 macrophages.

Nocodazole and latrunculin A treatment had no effect on the release of TNF-*α* by infected M1 macrophages (Figure 3(c)) or by uninfected and non-activated cells (Figure 3(d)). However, treatment with latrunculin promoted the inhibition of TNF-*α* release by 82.3% in uninfected M1 macrophages (Figure 3(d)). 

### 3.4. Effect of Latrunculin and Nocodazole on RAW 264.7 Macrophage Actin Organisation during Parasite-Host Interactions

**Figure 4:**
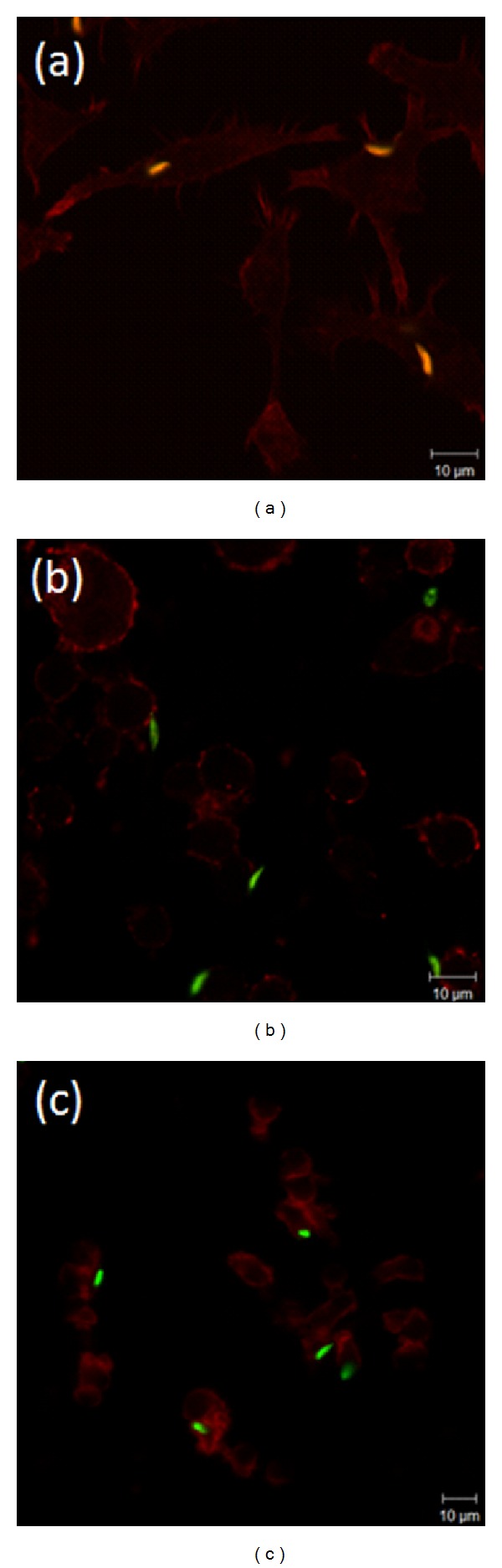
Integrity of the cytoskeleton influences the interaction between *L. braziliensis *and macrophages. Adherent Raw 264.7 macrophages were incubated for 10 min with (a) fresh DMEM, (b) 5 *μ*M latrunculin A to disrupt actin filaments, or (c) 5 *μ*M nocodazole to disrupt microtubules before exposure for 30 min to parasites that had been previously marked with CFSE. The panels are representative fields of colour-combined fluorescent images displaying parasites in green and F-actin (phalloidin staining) in red. Scale bars indicate imaging for all panels at 63x magnification.

The observed changes in the association indexes, NO production, and cytokine production prompted us to assess morphological changes in the macrophages. The actin cytoskeleton was stained in macrophages after interaction with *L. braziliensis* (Figure 4). In control macrophages without drug treatment, highly organised F-actin was observed, with prominent staining in the periphery of the cell and numerous instances of filopodial structures. In the representative image (Figure 4(a)), internalised parasites can be observed in orange, the result of the superposition of red and green staining, showing a colocalisation between actin cytoskeleton and the parasite, confirming the involvement of actin on this process as showed before. In the absence of actin filaments resulting from latrunculin A treatment (Figure 4(b)), the macrophages were observed to be much more round in shape and lacked filopodial structures. There were no cells with internalised parasites; instead, parasites can be observed, in green, attached to the macrophages or free in the extracellular milieu. Treatment with nocodazole to disrupt microtubules had a profound effect on the cell organisation of the macrophages. The organisation of stained F-actin was random, without prominent structures in the periphery of the cell and without the presence of filopodia. Similar to macrophages treated with latrunculin, there were no cells with internalised parasites, which can be observed in green attached to the macrophages. Some parasites appeared to be semi-internalised (Figure 4(c)).

### 3.5. Transfection of Macrophages with eGFP-DB (Myosin Va Tail)

**Figure 5:**
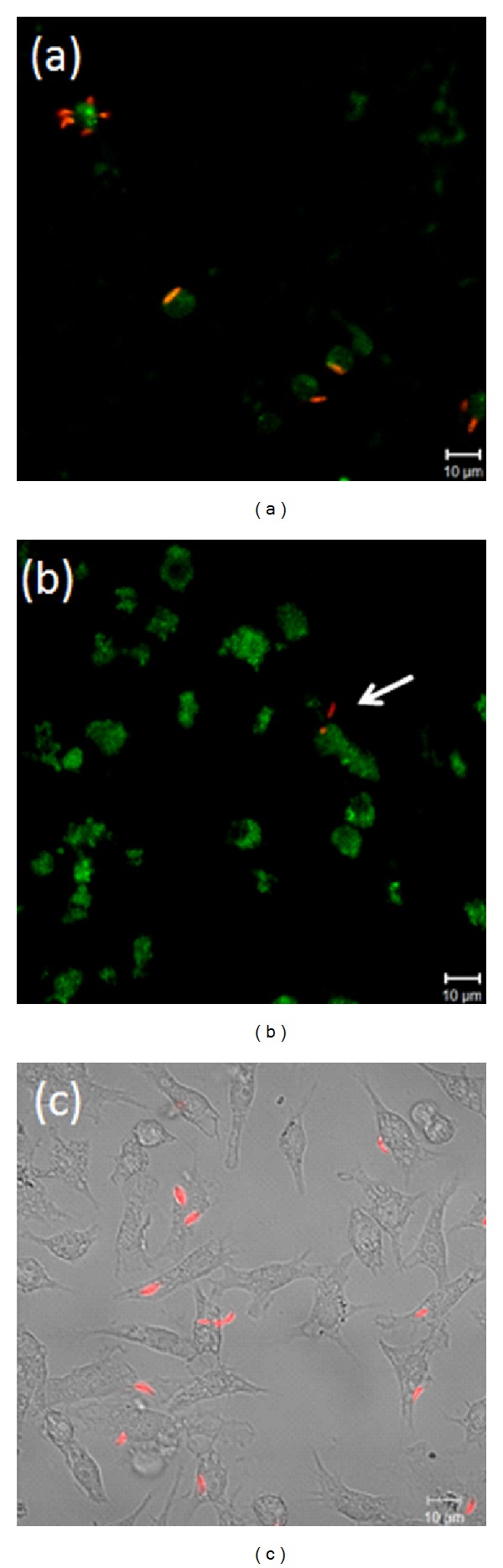
Internalisation of *L. braziliensis* requires myosin Va. The role of myosin Va was examined by observing the distribution of endogenous myosin Va in relation to parasites and the effect of the expression of a dominant negative tail domain fused to eGFP. Adherent Raw 264.7 macrophages were incubated with fluorescently marked parasites for 30 min before fixation, preparation and imaging by confocal microscopy. (a) shows endogenous myosin Va stained by an antibody (green) and parasites (red). (b) shows the myosin Va tail (green) and parasites (red). (c) shows mock-transfected macrophages by phase microscopy overlaid with a fluorescent image of parasites (red). Scale bars indicate imaging for all panels at 63x magnification.

Because of the high dependence on an intact cytoskeleton and the dynamic nature of the parasite/macrophage interactions, molecular motors were considered to be potentially important host cell factors. Myosin Va was the first candidate considered because of its wide range of expression and diverse functions, including involvement in phagosome movement. Using an antibody against MVa, we observed the presence of this protein in macrophages and the colocalisation of myosin with the parasite. Internalised parasites can be observed in orange, the result of the superposition of red and green staining (Figure 5(a)). To evaluate the role of myosin Va, a construct consisting of the myosin Va tail (brain isoform) fused to eGFP was transfected into macrophages before parasites were added. There was an abrupt decrease in the association index between transfected macrophages and *L. braziliensis* promastigotes (Figure 5(b)). The mock-transfected macrophages did not interfere with parasite interaction, and several promastigotes can be observed attached to or internalised by these cells (Figure 5(c)). The association index of the wild type macrophage was 53.25 ± 4.49 and that of the mock macrophages was 42.42 ± 3.91, while in the transfected macrophages, this index was 2.96 ± 0.94 (data not shown).

## 4. Discussion

The establishment of an *L. braziliensis* infection in humans, and the beginning of leishmaniasis, starts with the uptake of the parasite by macrophages recruited to the site of a sandfly bite [40]. As a professional phagocytic cell, the macrophage plays an important role in the immune response to foreign material, including pathogens. Normally, phagocytosis by macrophages initiates a destructive process that destroys the internalised microorganism, principally through NO production [41]. However, *Leishmania* has evolved mechanisms to neutralise the normal processes of macrophages, allowing it to survive within the cell and effectively avoid the immune system, thereby propagating the infection [39]. Understanding the pathways within the host cell that are modulated by the parasite has major implications for combating the consequences of an infection.

Considering the importance of phagocytosis, this study focused on the underlying cytoskeletal requirements, specifically F-actin and microtubules. Actin microfilaments, intermediate filaments, and microtubules are the major cytoskeleton components in eukaryotic cells. The cytoskeleton is a highly dynamic and responsive structure that is regulated by several proteins that are involved in a wide range of events such as signal transduction [42], cellular proliferation, cellular migration [29], phagocytosis and ROI, NO and cytokine production [43, 44]. Our initial experiments focused on the role of the cytoskeleton and the molecular motor myosin Va in the interaction between parasites and macrophages. Studies have shown the importance of the initial polymerisation of actin to the invasion of the host cells through phagocytosis [45–47] and the importance of interactions between the cytoskeleton and motor proteins such as myosin V [48].

Latrunculin A was chosen to depolymerise actin filaments because of its well-established ability to rapidly and reversibly modulate the organisation of actin in adherent cells [25, 49, 50]. In non-activated macrophages, the association index was significantly decreased after the reduction of F-actin (Figure 1). The index was also reduced in activated M1 macrophages. Although latrunculin A disrupts the actin cytoskeleton, at the concentration used in this work, some actin filaments still persist [51], which could explain the residual internalisation of* Leishmania*. The absolute index was greater in the activated macrophages than in non-activated macrophages, but the percent reduction from the nontreated controls was nearly equal. The observed higher index for the activated macrophages can be explained by the increase in phagocytosis that was promoted by LPS and IFN-*γ* treatment [52]. Despite the capacity of activated macrophages to kill parasites, the time frame of the experiments (30 min) was insufficient to observe any long-term effects.

The organisation of microtubules was disrupted through treatment with nocodazole [53, 54]. Nocodazole promoted high inhibition of parasite interactions with both M1 and non-activated macrophages, as measured by the association index. The effect was more pronounced in the M1 macrophages. This is in agreement with a previous observation that treatment of IFN-*γ*-activated RAW macrophages with nocodazole promoted the inhibition (40%) of phagocytosis indices [55]. Nocodazole was previously observed to inhibit by 40% the infection of intestinal epithelial cells (INT407) by *Enterobacter sakazakii* [56]. This low range of inhibition could explain the residual parasite-macrophage association observed in the presence of nocodazole. In addition, the nocodazole results suggest that the first steps in the internalisation of *Leishmania* by a macrophage are associated more with actin filaments than with microtubules.

It has long been observed that the treatment of macrophages with microfilament-inhibiting drugs such as cytochalasin D promotes a drastic decrease in *Leishmania *parasite binding. Both the number of parasites attached to macrophages and the proportion of infected macrophages diminish when macrophages were treated with 10 *μ*g/mL of this substance [57]. Based on studies using cytochalasin, the dissociation of parasite attachment from the subsequent entry into host cells has been reported since the 1970s in several parasitic protozoa, including *Leishmania *[58], *Trypanosoma *[59], and *Toxoplasma *[60].

In addition to phagocytosis interference, the possibility of these compounds to influence other microbiocidal macrophage events was tested. In this work, latrunculin has been shown to increase the NO production of non-activated and uninfected macrophages to the same extent as LPS and IFN-*γ* activation [44, 61, 62]. The monomeric form of actin (G-actin) promotes the stimulation of eNOS (endothelium NO synthase) [63]. In endothelial cells, NO production has been found to be directly related to changes in cell elasticity as a result of actin cytoskeleton reorganisation [64]. The increase in cell elasticity that occurs when actin is polymerised is accompanied by a decrease in NO production, while depolymerisation and, consequently, an increase in G-actin promote an increase in NO production [65].

Although the NO production of macrophages has primarily been associated with iNOS (induced NO synthase) activity, which is induced by the products of microorganisms such as LPS and a variety of inflammatory cytokines [66], RAW 264.7 mouse macrophages have been shown to constitutively express eNOS, and LPS-stimulation increased the activity of eNOS via changes in intracellular Ca^2+^ levels [67]. Because latrunculin associates with actin monomers, which prevent G-actin from polymerising [68], the accumulation of G-actin could be the key mechanism that causes the observed changes in the non-activated, uninfected macrophages. However, in the activated, infected macrophages, latrunculin promoted the inhibition of NO production. Likewise, activated macrophages treated with latrunculin or cytochalasin (another substance that disrupts the actin cytoskeleton) exhibited a decrease in NO production [69]. In contrast, nocodazole promoted an increase in the production of NO by infected M1 macrophages but inhibited NO production in uninfected macrophages (regardless of the activation status). Pulmonary artery cells (PAECs) treated with nocodazole have exhibited a decrease in NO production, but the exact mechanism remains unknown [53]. 

As already discussed, classical macrophage activation results in cells (M1) that are capable of producing high levels of tumour necrosis factor (TNF), reactive oxygen intermediates (ROIs), and iNOS expression. This activation is promoted by IFN-*γ*, and the pro-inflammatory response that is triggered results in the development of M1 macrophages. During infection by obligatory intracellular protozoan parasites such as *Leishmania*, *Trypanosoma*,* Toxoplasma,* and *Plasmodium*, these cells are necessary to control parasitemia, especially during the acute phase of the parasitosis [70–72]. However, depending on parameters such as host genotype, parasite virulence, and stage of infection, the hosts can also produce anti-inflammatory cytokines (Th2). These cytokines (IL-4 and IL-13) antagonise M1 macrophages, suppressing NO production by iNOS from L-arginine, and induce an alternative metabolic pathway of L-arginine catalysed by the enzyme arginase-1. This is the alternative pathway for macrophage activation, which involves type 2 responses and partly overlapping phenotypes, including IL-10 secretion. Therefore, these alternatively activated macrophages have been generically called M2 or AAM [10, 73, 74].

 Latrunculin and nocodazole were observed to inhibit IL-10 secretion in infected M1 macrophages, as well as in uninfected and non-activated macrophages. There were no significant changes measured in the uninfected M1 macrophages. TNF-*α* secretion by infected M1 macrophages was not affected by either treatment, and only latrunculin was able to inhibit TNF-*α* secretion in uninfected M1 macrophages. Because both actin and microtubules are involved in the establishment of cell polarity and the directed secretion of cytokines and cytolytic granules [74], the data suggest that interference with the cytoskeleton is primarily responsible for the inhibition of cytokine secretion.

Another possibility is the inhibition of cytokine production. Colchicine, a drug that is also capable of promoting the depolymerisation of microtubules, was observed to cause a strong reduction in the accumulation of LPS-induced TNF-*α* mRNA. This event suggests that a pretranslational effect may represent the primary mechanism by which colchicine reduces TNF-*α* production [75]. Similarly, latrunculin and nocodazole could affect the mRNA of these cytokines, especially considering the role of the cytoskeleton in mRNA localisation [76, 77].

In addition, the disruption of microtubules can be associated with cytokine signal transduction. Toll-like receptors (TLRs) are involved in proinflammatory cytokine production following the recognition of several pathogen-derived components. These receptors activate the conserved MyD88 pathway, triggering the transcription factors NF*κ*B and AP-1, which are essential factors in the production of inflammatory cytokines [78]. In dendritic cells, TLR2 and TLR4 are not present on the cell surface. They are associated with tubovesicular structures close to the Golgi complex and colocalise with microtubules. This suggests that TLR-decorated vesicles move along these structures. Supporting this, the depolymerisation of the microtubule network has been shown to disrupt the intracellular distribution of TLR2 and TLR4, which inhibits the production of IL-12 and TNF-*α* in response to *Neisseria meningitidis*. However, phagocytosis was not affected [79].

Myosin V motor proteins are responsible for cargo transportation and interact not only with actin filaments but also with several other components of the cytoskeleton, including microtubules, kinesins, and intermediate filaments [48]. Studies using RNA interference, gene deletion, and the expression of dominant-negative myosin tail constructs have been used to evaluate the role of this protein in the transport of motor organelles. These studies have shown a decrease in the speed or a total blockage of the cargo transport that is dependent on myosin Va [80–82]. Myosin Va appears to be involved in phagosome transportation, while other myosin classes appear to be involved in phagosome formation [24, 83, 84]. Araki [83] showed that myosin Va binds to the phagosome and F-actin, which restricts the movement of phagosomes. 

Here, the presence of myosin Va in macrophages was confirmed, and colocalisation was observed between myosin Va and *L. braziliensis* (Figure 5(a)). To characterise the involvement of myosin Va with *L. braziliensis*, macrophages were transfected with a myosin Va tail construct to generate a dominant-negative effect by disengaging myosin Va cargo from actin filaments and thereby interfering with transport. As shown in Figure 5(b), the presence of the myosin Va tail reduced the association between macrophages and *L. braziliensis*. This result suggests that myosin Va plays a role in the association of *L. braziliensis *with macrophages. 

Two important aspects of macrophage function that can be impacted by disrupting the cytoskeleton and myosin Va function are phagosome activity and receptor recycling. Recently, actin and microtubules were shown to have an important role in recycling from the phagosome [85]. In other cell types, myosin Va has been shown to contribute to membrane recycling and exocytosis [86, 87]. Considering the importance of the recycling of the mannose/fucose receptor (MFR) to the plasma membrane and its role in the ingestion of *L. donovani* [88], the decreased *L. braziliensis* association observed in dominant-negative myosin Va macrophages could be caused by the absence or reduction of important receptors such as MFR on the macrophage membrane. Further investigations are necessary to characterise the role of myosin Va in the association of *L. braziliensis *with macrophages.

Overall, the data clearly show the importance of F-actin, microtubules, and myosin Va during the interactions between macrophages and *L. braziliensis.* Furthermore, the observations of the changes in the production of NO, IL-10, and TNF-*α* suggest that modulation of the cytoskeleton could be a mechanism for *L. braziliensis* to overcome the natural responses of macrophages and establish an infection.
